# Myopericarditis After BNT162b2 mRNA Vaccination With Incidental Intramyocardial Bridging

**DOI:** 10.7759/cureus.34452

**Published:** 2023-01-31

**Authors:** Mohamed Elghazal, Inas M Alhudiri, Mohamed Said, Eiman Elhouderi, Adam Elzagheid

**Affiliations:** 1 Vaccination Unit, Department of Cardiology, Libyan Biotechnology Research Center, Tripoli, LBY; 2 Department of Genetic Engineering, Libyan Biotechnology Research Center, Tripoli, LBY; 3 Department of Internal Medicine, Beaumont Health, Dearborn, USA

**Keywords:** covid-19 vaccine, pfizer-biontech, bnt162b2, bnt162b2 mrna vaccine, coronavirus disease 2019, intramyocardial bridging, covid-19, mrna vaccination, pericarditis, myocarditis

## Abstract

Myocarditis and pericarditis are inflammatory conditions affecting the myocardium and pericardium, respectively. They are caused by infectious and non-infectious conditions, including autoimmune disorders, drugs, and toxins. Vaccine-induced myocarditis has been reported with viral vaccines, including influenza and smallpox. The BNT162b2 mRNA vaccine (Pfizer-BioNTech) has shown ‎great efficacy against symptomatic, severe coronavirus disease 2019 (COVID-19), hospital admissions, and ‎deaths.‎ The US FDA issued an emergency use authorization for the Pfizer-BioNTech COVID-19 mRNA vaccine for the prevention of COVID-19 in individuals ≥ five years. However, concerns were raised after reports of new cases of myocarditis following mRNA COVID-19 vaccines, especially among adolescents and young adults. Most cases developed symptoms after receiving the second dose. Here, we present a case of a previously healthy 34-year-old male who developed sudden and severe chest pain a week after the second dose of the Pfizer-BioNTech COVID-19 mRNA vaccine. Cardiac catheterization showed no angiographically obstructive coronary artery disease but it revealed intramyocardial bridging. This case report demonstrates that the mRNA COVID-19 vaccine can be associated with acute myopericarditis and the clinical presentation can mimic acute coronary syndrome. Despite that, acute myopericarditis associated with the mRNA COVID-19 vaccine is usually mild and can be managed conservatively. Incidental findings such as intramyocardial bridging should not exclude the diagnosis of myocarditis and should be carefully evaluated. COVID-19 infection has high mortality and morbidity even in young individuals, and all different COVID-19 vaccines were found effective in the prevention of severe COVID-19 infection and in decreasing COVID-19 mortality.

## Introduction

The US FDA issued an emergency use authorization (EUA) for the Pfizer-BioNTech coronavirus disease 2019 (COVID-19) mRNA vaccine for the prevention of COVID-19 in individuals ≥ five years [1]. The BNT162b2 mRNA vaccine (Pfizer-BioNTech) has shown ‎great efficacy against symptomatic, severe COVID-19 disease, hospital admissions, and ‎death [2].‎ However, concerns were raised after reports of new cases of myocarditis following mRNA COVID-19 vaccines, especially among adolescents and young adults [3]. Here, we present a case of a previously healthy 34-year-old male who developed myopericarditis following the second dose of his Pfizer-BioNTech COVID-19 vaccine.

## Case presentation

A 34-year-old male with no past medical history apart from heavy cigarette smoking presented to the emergency department of a private hospital in Tripoli, Libya with sudden onset left-sided stabbing chest pain approximately two hours after the pain had started. The pain was associated with shortness of breath and palpitations. The pain was severe enough to awaken him from sleep. The patient rated his pain 8/10 on a numerical scale rating. The pain was persistent and radiated to his left shoulder and back. The pain was aggravated by exertion and temporarily relieved by taking analgesics like paracetamol and aspirin tablets. The pain lasted for about two weeks but the severity decreased to 2-3/10 by the third day. The shortness of breath was aggravated by exertion and moderate activity. The patient received the second dose of the Pfizer-BioNTech COVID-19 vaccine a week before the onset of his symptoms. The patient had a previous history of respiratory symptoms suggestive of COVID-19 twice before he received the vaccine without a confirmed laboratory COVID-19 diagnosis. His latest illness was in four months prior to the administration of the COVID-19 vaccine and he had close contact with a family member who had a polymerase chain reaction (PCR)-confirmed diagnosis. The patient received the first dose of the Pfizer-BioNTech COVID-19 vaccine (September 2021) 21 days before ‎the second dose. He only had mild fatigue and pain at the site of injection for two days. After the second dose of the vaccine, he had more severe symptoms ‎with fatigue, pain at the site of injection, cough, and fever. The symptoms persisted for four days despite frequent paracetamol tablets. On presentation, vital signs were normal and physical examination was unremarkable.

**Figure 1:**
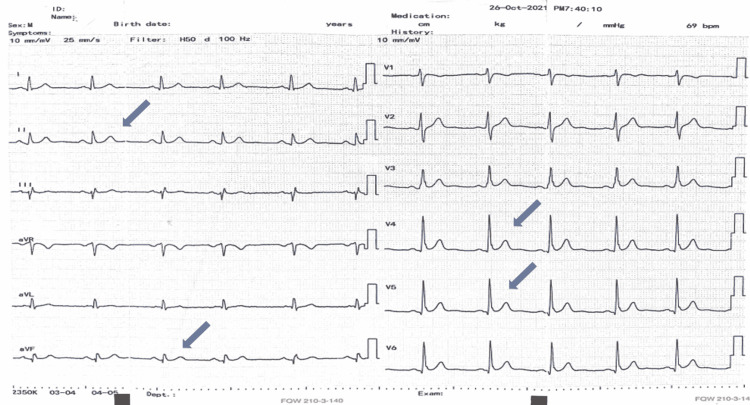
Twelve-lead electrocardiogram of the patient demonstrating ST elevation in septal, lateral, and leads.

ECG showed concave ST elevation in leads V4-V6 (septal and lateral walls) with no T wave inversion or reciprocal ST depression (Figure 1).

Initial cardiac enzymes showed slightly elevated creatine kinase-MB and normal troponin T. All other laboratory investigations were normal including markers of inflammation, renal and liver functions, electrolytes, and lipid profile (Table 1).

**Table 1:** Complete blood count and biochemical analysis results. CRP: C-reactive protein; CK-MB: creatine kinase-MB; HBA1C: hemoglobin A1c; HDL: high-density lipoprotein; LDL: low-density lipoprotein.

Parameter	Reference range	Patient
WBC (10^3^/µl)	4.5-10	7.4
RBC (millions/ µl)	3.5-5	4.7
Hemoglobin (g/dl)	11-15	13.5
Hematocrit (%)	42-52	39.9
Lymphocytes (%)	20-40	35.4
Neutrophils (%)	45-70	53.9
Platelets (10^3^/µl)	150-450	214
CRP (mg/L)	Up to 5	0.80
Troponin	<0.1	0.1
CK-MB	<25	44.9
Sodium (mmol/L)	136-144	140.4
Potassium (mmol/L)	3.5-5.4	4.01
Blood urea (mg/dl)	17-50	25
Serum creatinine (mg/dl)	0.70-1.20	1
Fasting blood sugar (mg/dl)	70-115	114
HBA1C (%)	4.8-5.6% = non-diabetic; 5.7-6.4% = pre- diabetic; >6.5% = diabetic	6.1
Triglyceride (mg/dl)	0-149	98
HDL cholesterol (mg/dl)	40-60	45.3
LDL cholesterol (mg/dl)	100-159	107.1
Bilirubin (total) (mg/dl)	0.3-1.2	0.7
Aspartate aminotransferase (U/L)	5-34	17
Alanine aminotransferase (U/L)	0-55	18
Anti-spike IgG antibodies after the second dose (AU/ml)	>10 AU/ml	312
Anti-spike IgG antibodies after the first dose	>10 AU/ml	34

The transthoracic echocardiogram was unremarkable. Because of chest pain and abnormal ECG, coronary angiography was performed and it demonstrated no angiographically significant coronary artery disease but it showed myocardial bridging (MB) localized in the middle portion of the left anterior descending coronary artery (Videos 1, 2).

**Video 1:** Day 1 left coronary angiography demonstrating no obstructive coronary artery disease. A myocardial bridge is seen in the middle portion of the left anterior descending artery.

**Video 2:** Day 1 left coronary angiography demonstrating the absence of arterial obstruction and ischemia. A myocardial bridge is seen in the middle portion of the left anterior descending artery.

Viral screens, including SARS-CoV-2, influenza A and B, and respiratory syncytial virus by reverse transcription-quantitative PCR, were all negative. The patient was managed for possible pericarditis with non-steroidal anti-inflammatory drugs and colchicine for chest pain and discharged home on hospital day two in stable clinical condition.

The patient was followed up for six weeks. His chest pain was improved and his ECG showed a resolution of ST elevation.

## Discussion

**Figure 2:**
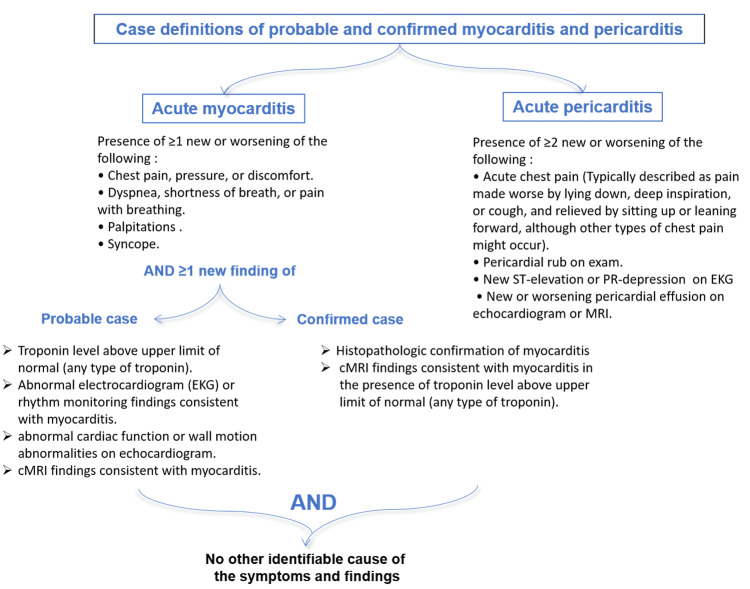
Case definitions of probable and confirmed myocarditis and pericarditis. Myopericarditis may be used for patients who meet the above criteria for both myocarditis and pericarditis. cMRI: cardiac magnetic resonance imagining.

The patient presented with new chest pain, shortness of breath, and palpitations along with ST abnormalities. The clinical course and investigations of our patient support the diagnosis of a probable case of acute myopericarditis according to the Centers for Disease Control and Prevention (CDC) working case definitions (Figure 2) [4].

We could not identify an alternative cause for the pericarditis other than the relationship to the timing of mRNA vaccination. Acute coronary syndrome was ruled out given the absence of obstructive coronary diseases on coronary angiography.

Myocarditis and pericarditis are inflammatory conditions affecting the myocardium and pericardium. They are caused by infectious (viral, bacterial, fungal, protozoal, helminth, and rickettsial) and non-infectious conditions, including autoimmune disorders, drugs, and toxins [5]. The most common clinical features are chest pain, dyspnea, fatigue, palpitations, ‎syncope, and cardiogenic shock. In young cases (aged < 35 years), sudden cardiac death can ‎occur. Fever, gastrointestinal disorders, and influenza-like symptoms were recorded in up to 80% of ‎patients with acute myocarditis [6].

Myocardial injury in viral myocarditis is mediated by triggering the immune system leading to lymphocyte infiltration into the myocytes and a cascade of virus-mediated cytotoxic events. This ultimately causes cardiomyocyte apoptosis and necrosis and induces the secretion of proinflammatory cytokines [7]. Autopsy reports of myocarditis following the COVID-19 Pfizer-BioNTech vaccination showed diffuse inflammatory infiltration, with neutrophil and histiocyte predominance within the myocardium and it was the cause of death in these patients [8,9]. Additionally, a study by Choi et al. described similar histological differences from other viral or immune-mediated myocarditis showing that the inflammatory infiltrates were predominantly neutrophils and histiocytes, rather than lymphocytes [10].

Given the smoking history and chest pain, acute coronary syndrome was an important differential diagnosis. However, the absence of other cardiovascular disease risk factors, regional wall motion abnormalities, angiographically significant coronary artery disease, and the time interval from the COVID-19 vaccine make pericarditis with probable myocarditis more likely than acute ST-elevation myocardial infarction.

A Danish study found that myocarditis associated with mRNA vaccination was a rare event with mild symptoms and no mortality even in young age groups (12-39 years). The study also showed that SARS-CoV-2 infection was associated with a 14-fold increased risk of cardiac arrest or death in the 28-day post-infection compared with uninfected [11]. Another study demonstrated an increased risk of myocarditis associated with mRNA vaccination within a week of receiving the first or second dose, the risk being more associated with the mRNA-1273 vaccine than the BNT162b2 vaccine [12].

In a case series of eight patients with myocarditis after mRNA vaccination, all individuals were males between the ages of 21 and 56 years. Five patients had regional wall motion abnormalities with inferior and inferolateral walls involved. The patients presented with symptoms of acute myocarditis three days after the second dose [13]. Reports received by the Canadian Adverse Events found statistically significant higher rates of myocarditis and/or pericarditis following the second dose of mRNA-1273 vaccination than BNT162b2 vaccination in males aged 18-29 years followed up to seven days post-vaccination [14]. The risk of myocarditis is significantly higher when the time between the two doses is ≤30 days compared with ≥56 days [15].

This case report discussed a case of myopericarditis presented with ST elevation incidentally found to have MB on coronary angiography. The incidence of MB was cited as an average of 25% based on autopsy studies [16,17]. On the other hand, the angiographic prevalence of MB was reported as 5% [18]. Although MB has been reported to be associated with acute coronary syndrome in many studies, no definite pathologic correlation between MB and acute coronary syndrome has been clearly established and it is generally considered a benign condition [19,20].

## Conclusions

This case report demonstrates that the mRNA COVID-19 vaccine can be associated with acute myopericarditis and the clinical presentation can mimic acute coronary syndrome. Despite that, acute myopericarditis associated with the mRNA COVID-19 vaccine is usually mild and can be managed conservatively. COVID-19 infection has high mortality and morbidity even in young individuals and all different COVID-19 vaccines were found effective in the prevention of COVID-19 infection and decreasing COVID-19 mortality.
